# Abdominal imaging utilization in the emergency department: trends over two decades

**DOI:** 10.1186/1865-1380-4-19

**Published:** 2011-04-27

**Authors:** Ali S Raja, Koenraad J Mortele, Richard Hanson, Aaron D Sodickson, Richard Zane, Ramin Khorasani

**Affiliations:** 1 Center for Evidence-Based Imaging, Brigham and Women's Hospital, 75 Francis St., Boston, MA, USA; 2 Department of Emergency Medicine, Brigham and Women's Hospital, 75 Francis St., Boston, MA, USA; 3 Department of Radiology, Brigham and Women's Hospital, 75 Francis St., Boston, MA, USA; 4 Harvard Medical School, Boston, MA, USA

## Abstract

**Background:**

To assess patterns of use of abdominal imaging in the emergency department (ED) from 1990 to 2009.

**Methods:**

We retrospectively reviewed data on adult ED patients treated between 1990 and 2009 at our university-affiliated quaternary care institution. Examinations were coded by abdominal imaging modality: x-ray, sonography, CT, or MRI. Proportional costs for each imaging modality were evaluated using relative value units (RVUs). Chi-square tests were used to assess for significant trends.

**Results:**

The intensity of abdominal imaging per 1,000 ED visits increased 19.3% from 1990-2009 (*p *= 0.0050). The number of abdominal CT scans per 1,000 ED visits increased 17.5-fold (*p *< 0.0001). Similarly, the number of abdominal MRIs per 1,000 ED visits increased from 0 to 1.0 (*p *< 0.0001), and the number of abdominal sonographs per 1,000 ED visits increased 51.6% (*p *= 0.0198). However, the number of x-ray examinations per 1,000 ED visits decreased 81.6% (*p *< 0.0001). Abdominal imaging RVUs per 1,000 ED visits increased 2.7-fold (*p *< 0.0001), due primarily to CT imaging, which accounted for 14% of RVUs in 1990 and 76% of RVUs in 2009.

**Conclusions:**

The intensity of abdominal imaging examinations per 1,000 ED visits and the number of abdominal imaging RVUs increased significantly over a 20-year period. CT replaced x-ray as the most common abdominal imaging modality for evaluation of ED patients. In light of these increasing costs as well as the increased radiation exposure of CT, clinical decision rules and computerized decision support may be needed to ensure appropriate utilization of abdominal CT in the ED.

## Background

Over the past two decades the availability and technological capability of radiologic imaging has increased worldwide [1]. While this increased availability of advanced imaging has been associated with improved patient outcomes for some diseases [2], there are growing concerns about the possibility of inappropriate utilization of imaging because of its potential contribution to both patient harm [3,4] and rising health care costs [5,6]. Long-term observational studies of imaging use are necessary to gain a better understanding of utilization patterns and trends in order to focus research into the appropriateness of abdominal imaging upon the areas of highest use and fastest growth [7].

Acute abdominal pain is one of the most common chief complaints in adults presenting to the ED [8]. It is also a symptom with a broad differential diagnosis [9], and a number of imaging modalities can be used to assist in elucidating its cause [10]. Three studies of trends in ED abdominal imaging utilization have been published in the past decade [11-13]. However, all three were limited in their time span as well as their focus on specific imaging modalities. Broder et al. found that abdominal CT use increased 72% from 2000 to 2005 and Lee et al. found that abdominal CT use increased 150% from 2001 to 2007, but neither study evaluated these increases in the context of other abdominal imaging modalities [11]. Pines evaluated multiple modalities and found that both abdominal CT and abdominal ultrasound increased between 2001 and 2005, but his study's time span was relatively brief [12]. Outside of the ED, Levin et al. analyzed general trends in abdominal imaging and noted an increase in total abdominal imaging as well as the proportion of CT and ultrasound examinations between 1996 and 2005 [14]. However, the applicability of these data to an ED population is unknown as no prior study has specifically examined the long-term trend in ED abdominal imaging.

Therefore, the objective of this study was to assess the utilization of ED abdominal radiology services at our institution over two decades and to determine whether the trends in CT imaging noted in smaller studies were observed over this longer time period. To assess changes in the proportional cost of abdominal imaging related to each imaging modality, we also evaluated trends in relative value units (RVUs) over this time period.

## Methods

The population for this institutional review board-approved study included all adult patients visiting the ED of our hospital between 1 January 1990 and 31 December 2009. Our facility is a 777-bed university-affiliated quaternary care hospital with approximately 60,000 adult ED patient visits per year and nearly 7,000 abdominal imaging procedures per year. All abdominal radiological studies performed for ED patients were identified using the hospital's clinical radiology database, and those studies coded for by exam codes specific for abdominal imaging were included in the study. These studies were classified by modality: x-ray, sonography, CT, and MRI, and then normalized per 1,000 ED visits. Fluoroscopy, nuclear medicine, and interventional procedures were excluded as they are not performed in the ED.

To measure changes in proportional charges due to each modality, we also analyzed RVUs normalized per 1,000 ED visits. We retrospectively applied the January 2010 current procedural terminology (CPT) code-specific RVUs from the Centers for Medicare and Medicaid Services (CMS) for each type of abdominal imaging study to the entire data set in order to produce a consistent scale for assessing relative changes throughout the period. CMS publishes a Physician Fee Schedule listing fees for over 7,000 physician services, including imaging examinations. These fees are measured by RVUs and broken into three categories: Work RVUs (which account for physician time and technical skill), Facility RVUs (which account for non-physician time as well as building space and equipment), and Liability RVUs (which account for the cost of malpractice insurance premiums). For this analysis, Liability RVUs were grouped under Facility RVUs because of the small proportion of Liability RVUs assigned to imaging procedures and we analyzed Work, Facility, and Total Imaging RVUs per 1,000 ED visits.

Chi-square tests for trend, using SAS 9.1 (SAS Institute Inc., Cary, NC), were performed in order to determine whether time was a significant predictor of the number of abdominal imaging studies as well as the number of RVUs, both per 1,000 ED visits. A *p*-value < 0.05 implied that the observed trend was significant over time.

## Results

**Figure 1:**
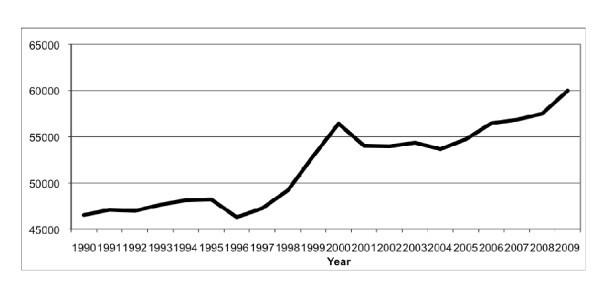
**Annual number of ED visits**.

**Figure 2:**
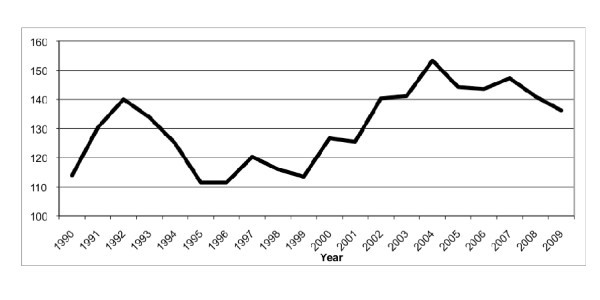
**Number of abdominal imaging studies per 1,000 ED visits**.

From 1990 to 2009, the total number of ED visits per year increased 28.9%, from 46,534 to 59,982 (Figure 1), and the number of abdominal imaging studies per 1,000 ED visits increased 19.3%, from 114 to 136 (*p *= 0.005) (Figure 2).

**Figure 3:**
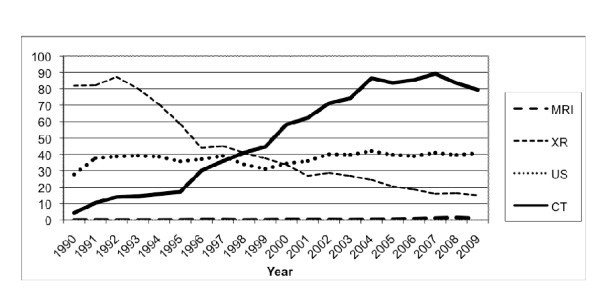
**Annual number of abdominal imaging studies per modality per 1,000 ED visits**.

The annual number of abdominal CT scans per 1,000 ED visits increased 17.5-fold, from 4.3 in 1990 to 79.4 in 2009 (*p *< 0.0001) (Figure 3). Similarly, the number of abdominal MRIs per 1,000 ED visits increased from 0 in 1990 to 1.0 in 2009 (*p *< 0.0001) and the number of abdominal sonography examinations per 1,000 ED visits increased 51.6%, from 27 to 41 (*p *= 0.0198). During the same 20-year period, the number of abdominal x-rays per 1,000 ED visits decreased 81.6%, from 81.9 to 15.1 (*p *< 0.0001).

**Figure 4:**
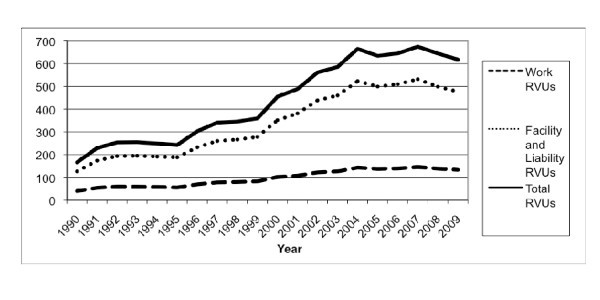
**Relative Value Units (RVUs) per 1,000 ED visits**.

**Figure 5:**
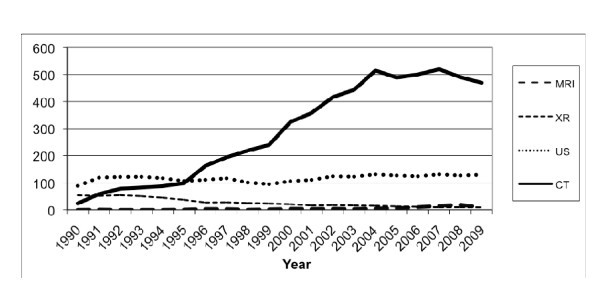
**Relative Value Units (RVUs) per modality per 1,000 ED visits**.

Work RVUs per 1,000 ED visits increased 2.3-fold (40.2 to 133.2, *p *< 0.0001) and facility RVUs per 1,000 ED visits increased 2.8-fold (126.6 to 476.7, *p *< 0.0001), leading to a 2.7-fold increase in total imaging RVUs per 1,000 ED visits (166.1 to 617.8, *p *< 0.0001) (Figure 4). The total RVUs per 1,000 ED visits attributable to ED abdominal CT imaging increased 18.9-fold, from 23.6 in 1990 to 469.4 in 2009 (*p *< 0.0001) (Figure 5). Similarly, the total RVUs per 1,000 ED visits attributable to ED abdominal MRI increased from 0 to 9.5 (*p *< 0.0001), and the total RVUs attributable to ED abdominal sonography increased 46.4% (88.5 to 129.6, *p *= 0.0084). However, the total imaging RVUs per 1,000 ED visits attributable to x-ray imaging decreased 82.8% over the period, from 53.9 in 1990 to 9.3 in 2009 (*p *< 0.0001).

**Figure 6:**
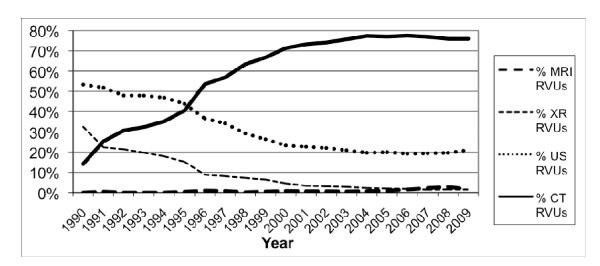
**Proportion of Relative Value Units (RVUs) attributable to each imaging modality**.

These trends in imaging RVUs led to a 4.4-fold increase in the proportion of ED abdominal imaging RVUs attributable to CT imaging, which rose from 14.2% in 1990 to 76.0% in 2009 (*p *< 0.0001) (Figure 6), as well as an increase in the proportion of ED abdominal imaging RVUs attributable to MRI imaging, which rose from 0% in 1990 to 1.5% in 2009 (*p *< 0.0001). Conversely, the proportion of ED abdominal imaging RVUs attributable to x-ray decreased 95.4% (32.5% in 1990 to 1.5% in 2009, *p *< 0.0001), while the proportion attributable to ED abdominal sonography decreased 60.6% (53.3% in 1990 to 21.0% in 2009, *p *< 0.0001).

## Discussion

The intensity of abdominal imaging, measured as the number of abdominal imaging studies performed per 1,000 ED visits, increased significantly during our 20-year study period. However, the imaging modality of choice shifted, as CT replaced x-ray as the preferred method of imaging the abdomen in ED patients. Additionally, the total RVUs attributable to abdominal imaging per 1,000 ED visits almost quadrupled during the study period and were most impacted by the 18.9-fold increase in RVUs per 1,000 visits attributable to abdominal CT scans.

When compared with data from 1990, the fraction of abdominal imaging RVUs accounted for by CT and MRI has risen 4.5-fold. This increase is not surprising in light of the rapidly advancing clinical uses for these modalities [15] as well as the increased quality of care and measured outcomes sometimes associated with their use [2]. However, given both the clinical risks [16] and the high cost of these studies [17], efforts at controlling expenditures should begin with identifying areas of highest use, followed by the implementation of strategies for minimizing the inappropriate use of imaging resources [18]. These strategies will need to be informed by research on the yield of different imaging techniques in specific clinical scenarios and the impact of imaging practices on clinical decision-making, therapy, and patient outcomes [19]. While this analysis has been done for CT utilization in other areas of the body (notably head CT in minor head injury [20] and chest CT for the evaluation of pulmonary embolism [21]) and other abdominal imaging modalities in the ED (including both x-ray [22] and sonography [23]), it has not yet been done for ED abdominal CT. Our results clearly confirm that CT has become the predominant method of imaging ED patients at our institution and that it should be the focus of future studies to help guide evidence-based strategies for abdominal imaging. These studies are necessary in order to develop criteria for appropriate testing by organizations such as the American College of Emergency Physicians and the American College of Radiology, since the Affordable Care Act of 2010 will rely on these criteria to vary "payment to physicians who order advanced diagnostic imaging services [CT, MRI and nuclear medicine] according to the physician's adherence to appropriateness criteria of such services" [24].

While this study did not assess the appropriateness of abdominal imaging practices, it did demonstrate that total adjusted abdominal imaging rates among ED patients are increasing and that the increased use of CT and MRI has been associated with a marked decline in the use of conventional studies. The utilization of abdominal imaging resources in the ED is determined primarily by the practices of the emergency physicians who order these procedures, and the use of validated decision rules has been shown to impact the utilization of imaging in other body areas. Most recently, Stiell et al. demonstrated that use of their Canadian C-Spine Rule led to a 12.8% relative reduction in ED cervical spine imaging [25]. A clear need exists for similar decision support tools to aid clinicians in determining the need, and best modality, for abdominal imaging in the ED. The development of these tools will likely lead to reportable quality measures regarding their use, allowing for the determination and comparison of measure compliance and a decrease in the potential overutilization of abdominal imaging.

Our study has several limitations. First, we studied only ED data and cannot comment on either inpatient or outpatient abdominal imaging utilization or cost. Second, our study may not have accurately measured the true utilization rate of abdominal imaging studies per ED visit, since we only analyzed data on imaging performed at our institution. Any abdominal imaging performed at referring hospitals prior to transfer to our Level 1 trauma center was not captured in this study. Third, we studied data from only one quaternary care hospital, and the results may not be generalizable to other institutions or regions. Fourth, we retrospectively applied the most recent RVUs for each imaging CPT code to data from all years in the study. While this is admittedly suboptimal, it was necessary in order to establish a consistent scale with which we could assess changes in the relative costs. Finally, our study looked only at overall trends in abdominal imaging and did not analyze patterns for specific clinical indications. Further studies will be required to identify trends regarding specific diagnoses in order to both better understand usage patterns and to develop guidelines for appropriate use of specific abdominal imaging modalities.

## Conclusions

The total number of abdominal imaging procedures per 1,000 ED visits at our institution has increased over the past 2 decades, and the proportion of CT and MRI studies has risen significantly. This increase in the utilization of more expensive imaging modalities led to a significant increase in the RVUs attributable to ED abdominal imaging. Future research efforts should be directed towards developing clinical decision tools and appropriateness criteria for abdominal imaging.

## Competing interests

The authors declare that they have no competing interests.

## Authors' contributions

ASR and RK conceived of the study; ASR, KJM, RH, ADS, RZ, and RK participated in its design and coordination and helped draft the manuscript. All authors read and approved the final manuscript.
